# Prediction model for rectal temperature in cats with different baseline characteristics using a non-contact infrared thermometer

**DOI:** 10.14202/vetworld.2024.2193-2203

**Published:** 2024-10-04

**Authors:** Nattakarn Naimon, Thitichai Jarudecha, Metita Sussadee, Rattana Muikaew, Supochana Charoensin

**Affiliations:** 1 Department of Veterinary Nursing, Faculty of Veterinary Technology, Kasetsart University, 50 Ngam Wong Wan Road, Ladyaow, Chatuchak, Bangkok, Thailand; 2 Department of Veterinary Technology, Faculty of Veterinary Technology, Kasetsart University, 50 Ngam Wong Wan Road, Ladyaow, Chatuchak, Bangkok, Thailand

**Keywords:** cat, noncontact infrared thermometer, prediction model, rectal temperature

## Abstract

**Background and Aim:**

Body temperature is the most useful clinical parameter for evaluating animal health. In clinical practice, rectal temperature is the gold standard for assessing body temperature, but rectal temperature measurement is not convenient and can cause stress in animals. The non-contact infrared thermometer is considered an alternative method for skin temperature measurements in animals. Many biological factors may influence the response of body regions to thermal challenges; thus, the identification of these variables is essential for accurate infrared temperature measurements. This study aimed to estimate the relationship between the physiological factors of cats and their body temperature measured across various body positions, as well as to propose a model for predicting rectal temperature using an infrared thermometer.

**Materials and Methods:**

A total of 184 client-owned cats were included in this study. The infrared temperature (°F) was measured using a non-contact infrared thermometer at five body positions: maxillary canine gingival margin (GCT), anal skin (ANS), inguinal canal (ING), ear canal (EC), and palmar pad. The five biological factors (age, body condition score [BCS], gender, hair type, and hair color) were recorded and analyzed to adjust predictive factors for rectal temperature prediction. All statistical analyses were performed using multivariable linear regression. The rectal temperature prediction model was then designed using the forward stepwise selection method.

**Results:**

Based on multivariable linear regression analysis of infrared temperature results, the pre-prediction model showed significant correlations with rectal temperature for ANS, GCT, and EC (p = 0.0074, 0.0042, and 0.0118, respectively). Moreover, the combination of infrared temperatures on ANS and ING was the most appropriate parameter for predicting rectal temperature (p = 0.0008). All models were adjusted according to the baseline characteristics of the cats. However, the adjusted R-squared values of the pre-prediction model of the infrared temperature on the ANS, GCT, and EC and the final prediction model by the infrared temperature on the ANS combined with the ING were low (8.7%, 8.9%, 7.3%, and 12.8%, respectively).

**Conclusion:**

The prediction model of rectal temperature of cats by infrared temperature from a non-contact infrared thermometer in ANS combined with ING and adjusted by age, BCS, hair type, and hair color may be applicable for use in clinical practice. This study found that the adjusted R-squared values of all models were low; the predictive model will need to be developed and used to test validity and reliability with an external study group for assessing their practical usefulness.

## Introduction

Body temperature measurement is the most useful method for clinically evaluating animal health. Abnormal temperature changes can indicate the animal’s basic physical condition, such as infection or shock [1]. Various methods are used for measuring body temperature, such as rectal thermometers, infrared tympanic thermometers, and thermal sensing microchips [2]. In clinical practice, the body temperature of animals is measured using a rectal thermometer, but it has been found that measuring temperature is not convenient, especially when the animal is aggressive or sick with problems in the pelvic area or the rectum and anus area (recto-anal) [3]. This method can also cause stress in animals. Measurements take time and have a high chance of infecting animals and can cause injury to both the animal and the veterinarian [4]. It was found that the measured temperature was lower than the actual temperature because the mercury tip was in contact with feces or gas within the intestines [5].

A digital thermometer was developed to measure body temperature based on the problem of mercury being used to measure rectal temperature [6]. However, there are few other methods that can measure temperatures as close to rectal temperature [7]. The method used in humans is to measure body temperature using infrared rays (infrared thermometer). A non-contact infrared thermometer has been used a lot during the pandemic of severe acute respiratory syndrome coronavirus 2 to measure infrared radiation on the skin or forehead [8, 9]. This method has received interest in animal applications as well. A previous study by Mota-Rojas *et al*. [10] on cats found that infrared thermography seems to be a promising, useful, easy, non-invasive, and rapid method for detecting aortic thromboembolism in cats, particularly in emergencies and proved to be a sensitive and effective method for detecting temperature differences between malignant and benign skin tumors in cats [11] and injury areas in dogs [12]. Thermal images obtained through infrared measurements can reduce animal stress, which may have a negative effect on animal welfare and trigger several behavioral changes, especially in cats [13]. A previous study by Giannetto *et al*. [14] on infrared temperature in cats found that infrared temperature in the eye is significantly related to rectal temperature and sensitive to accurately detect changes associated with rectal temperature. A study of 29 cats found that the infrared temperature measurement method on the ear canal (EC) can be used to measure temperature, with results consistent with rectal temperature [15]. However, previous studies on other animals found that the infrared temperature of the EC is significantly different [1] and has less correlation with rectal temperature [16]. The same as the study of cutaneous temperature using a digital infrared camera and a non-contact infrared thermometer did not reflect rectal temperature in goats [17]. However, animals’ body temperature varies according to the measurement location [18] and size [19]. A previous study by Kwon and Brundage [20] on dogs has found that the breed affects the temperature level of the skin. Long-haired dogs have a lower skin temperature than thin and short-haired dogs. Larger breeds have higher body temperatures than smaller breeds [21]. In addition, other factors such as environmental temperature, body weight, hair density, gender, and age also affect the relationship between rectal and skin temperatures in animals [3]. The body’s skin temperature depends on synergistic properties arising from peripheral blood flow. This is the interaction between the surface and the external environment, such as the thermal properties of hair coats [22, 23]. Biological factors may strongly influence the thermal responses of body regions [24]. Moreover, the identification of these variables is necessary to obtain accurate results [25].

This study aimed to analyze the relationship between the characteristic factors of cats and infrared temperature measured at various positions on the body and to design a model to predict rectal temperature using infrared temperature after adjusting for characteristics that could be confounding variables. This study provides information for future studies in developing a clinical device to measure animal body temperature using infrared radiation.

## Materials and Methods

### Ethical approval

This study was approved by the Animal Ethics Committee of Kasetsart University (Approval no. ACKU64-VTN-015) Bangkok, Thailand.

### Study period and location

This study was conducted from August 2019 to December 2021 at two animal hospitals in Bangkok, Thailand.

### Animals

The selection of cats was determined by census, taking into consideration the rule of thumb technique for sample size calculation [26]. The sample size was 180 cats (30 cats per 1 predictor) and 2% had missing data; thus, 184 cats were recruited for the study. The inclusion criteria for cats in this study were age >2 months, normal skin, normal gingival, and not having otitis. Cats that were aggressive and/or in severe conditions of another disease were excluded.

### Experimental design

The study design was cross-sectional, and data collection was performed using cohort approach techniques. Five predictor areas on the body and five factors of cat characteristics were used to predict rectal temperature. The predictor areas were infrared temperature of the maxillary canine gingival margin (GCT), anal skin (ANS), inguinal canal (ING), ear canal (EC), and palmar pad (PAL), and five factors were age, body condition score (BCS), gender, hair color, and hair type.

On arrival at the animal hospitals, each cat was recorded for the (BCS, 5 scores), gender, weight, breed, hair color, hair type (between long-, short-, and non-hair), age, infrared temperature in five areas, and rectal temperature. The hair colors were divided into eight groups (non-hair, solid, pattern, and point). The solid color groups were white, black, blue, cream, and red [27]. Following the collection of data, the age of cat was considered to be separated into four groups, which were 0–1 year, 1–7 years, 7–10 years, and more than 10 years. The division of the cat’s lifespan was referenced in the 2021 American Animal Hospital Association/American Association of Feline Practitioners Feline Life-Stage Guidelines [28].

The measurement of rectal and infrared temperatures was conducted in the examination room with air conditioning control at 24°C–25°C. After the cat had rested with its owner in the waiting area for 5–10 min. The mercury thermometer used in this study is a long cylindrical bulb type. The infrared temperature (°F) was measured using a non-contact infrared thermometer (Model UX-A-03, Guangdong Food and Drug Supervision Equipment Production License 20204135, China). In the clinical examination room, the examination table was covered with a dark-colored towel. The thermometer was placed at 2 cm from the predictor areas, three measurements per area to avoid possible ambient light sources. Finally, the rectal temperature (°F) was measured using a rectal thermometer for 1 min. The same operator performed all measurements to improve the reproducibility of the experiment.

### Statistical analysis

The data were analyzed using the STATA software package (version 17.0, Stata Corporation, Texas, USA). The Shapiro–Wilk test was applied to evaluate data obtained from the rectal and non-contact infrared thermometers. The mean (± Standard deviation) was normally distributed, whereas the median and interquartile range were non-normally distributed for every baseline characteristic of cats. The baseline characteristics of the cats are presented as numbers and percentages. The univariate analysis of baseline characteristics and predictor area was performed using simple linear regression analysis. The baseline characteristic, which was considered statistically significant at a p ≤ 0.05, and the predictor area with a p ≤ 0.2 were used for multivariable analysis to design a pre-prediction model of real temperature. The predictor area was then processed to forward stepwise regression (p ≤ 0.1) for multivariable analysis to design the final prediction model. The final prediction model was then analyzed by multivariable linear regression between predictor areas, baseline characteristics, and rectal temperature. The difference was considered statistically significant if p ≤ 0.05. The multivariable fractional polynomial approach was used to analyze the linear correlation of continuous variables. The adjusted R-squared value is shown in any graph; it is the coefficient of determination, which provides information about the goodness of fit of the model.

## Results

The breeds of cats included Domestic short-hair (n = 109), Scottish Fold (n = 32), Persian (n = 20), British Shorthair (n = 7), American Shorthair (n = 4), Siamese (n = 3), Maine Coon (n = 2), Sphynx (n = 2), American Curl (n = 1), Korat (n = 1), Munchkin (n = 1), Ragdoll (n = 1), and Suphalak (n = 1). The baseline characteristic data and the temperatures of five predictor areas and the rectum were measured in 184 cats. There were 182 cats with complete data and two cats with incomplete data on age. Table-1 shows the mean infrared temperatures recorded in the five areas using three measurements. However, a non-normal distribution of infrared temperature was found in each region based on baseline characteristic analysis. 82 cats were neutered (44.6% of total cats). The sterilization did not affect rectal and infrared temperatures after statistical analysis (data not shown).

**Table-1:** The baseline characteristic of cats with rectal temperature (°F) and infrared temperature (°F) from predictor areas of cats.

Variables	n (%)	Mean (SD)	Median (IQR)				
	
		Rectal	GCT	ANS	ING	EC	PAL
Gender							
Female	109 (59.2)	101.00 (0.99)	98.13 (97.87, 98.60)	98.00 (97.77, 98.73)	97.43 (97.10, 97.67)	98.01 (97.83, 98.67)	97.03 (96.80, 97.30)
Male	75 (40.8)	100.97 (1.08)	98.20 (97.87, 98.70)	97.93 (97.67, 98.40)	97.17 (96.87, 97.10)	98.00 (97.77, 98.80)	96.90 (96.77, 97.23)
Age							
0–1 year	108 (59.3)	101.22 (0.95)	98.23 (97.93, 98.87)	98.20 (97.77, 98.83)	97.37 (96.90, 97.72)	98.10 (97.80, 98.90)	97.02 (96.73, 97.30)
1–7 years	63 (34.6)	100.71 (1.07)	98.00 (97.80, 98.37)	97.93 (97.73, 98.20)	97.37 (97.03, 97.57)	98.00 (97.73, 98.53)	96.90 (96.80, 97.23)
7–10 years	7 (3.8)	100.94 (0.95)	98.57 (98.17, 99.27)	97.77 (97.50, 97.90)	97.67 (97.10, 97.93)	98.33 (98.00, 99.50)	97.23 (97.03, 97.30)
>10 years	4 (2.2)	99.8 (0.54)	98.15 (97.78, 98.95)	97.72 (97.65, 97.92)	97.07 (96.87, 97.55)	98.00 (97.92, 98.07)	97.13 (96.07, 97.20)
BCS							
Score <3	17 (9.2)	100.82 (1.37)	98.13 (97.87, 98.60)	98.13 (97.77, 98.83)	97.45 (97.10, 97.77)	98.02 (97.77, 98.68)	97.03 (96.82, 97.30)
Score 3	104 (56.5)	101.05 (0.99)	98.00 (97.87, 98.33)	97.77 (97.47, 98.07)	97.43 (97.30, 97.63)	98.00 (97.67, 98.33)	97.10 (96.80, 97.23)
Score >3	63 (34.2)	100.95 (0.98)	98.27 (97.93, 98.93)	97.93 (97.70, 98.33)	97.17 (96.83, 97.50)	98.17 (97.87, 98.90)	96.90 (96.67, 97.17)
Hair type							
No hair	2 (1.1)	100.30 (0.14)	98.06 (97.87, 98.27)	98.27 (97.93, 98.60)	99.03 (98.63, 99.43)	98.67 (97.67, 99.67)	97.08 (96.40, 97.77)
Short hair	158 (85.9)	100.98 (1.00)	98.15 (97.87, 98.67)	98.00 (97.77, 98.60)	97.43 (96.93, 97.70)	98.08 (97.83, 98.73)	97.03 (96.80, 97.30)
Long hair	24 (13.0)	101.13 (1.20)	98.15 (97.97, 98.62)	97.62 (97.42, 98.13)	97.10 (96.83, 97.27)	97.93 (97.65, 98.22)	96.97 (96.77, 97.10)
Hair color							
White solid	18 (9.8)	100.92 (0.83)	98.03 (97.67, 98.40)	97.78 (97.37, 98.20)	97.20 (96.70, 97.70)	97.95 (97.67, 98.27)	96.77 (96.53, 97.03)
Black solid	28 (15.2)	101.07 (1.06)	98.03 (97.87, 98.42)	98.00 (97.67, 98.57)	97.20 (96.92, 97.47)	97.97 (97.73, 98.53)	96.90 (96.68, 97.20)
Blue solid	17 (9.2)	101.18 (0.92)	98.23 (97.87, 99.03)	97.93 (97.70, 98.13)	97.10 (96.73, 97.50)	97.83 (97.53, 98.50)	96.90 (96.73, 97.23)
Cream solid	10 (5.4)	101.30 (0.81)	98.33 (98.00, 98.67)	98.20 (97.80, 98.40)	97.43 (97.03, 97.87)	98.45 (97.73, 98.80)	97.08 (96.90, 97.23)
Red solid	26 (14.1)	101.00 (1.22)	98.23 (97.87, 98.57)	97.95 (97.73, 98.57)	97.33 (96.90, 97.70)	98.38 (98.00, 99.03)	97.10 (96.87, 97.17)
Patterns	62 (33.7)	100.92 (1.10)	98.18 (97.97, 98.77)	98.17 (97.77, 98.73)	97.43 (97.10, 97.73)	98.12 (97.90, 98.73)	97.10 (96.87, 97.37)
Points	21 (11.4)	100.92 (0.88)	98.13 (97.80, 98.27)	97.93 (97.83, 98.77)	97.43 (97.10, 97.50)	98.00 (97.93, 98.60)	96.90 (96.73, 97.23)
No hair	2 (1.1)	100.30 (0.14)	98.07 (97.87, 98.27)	98.27 (97.93, 98.60)	99.03 (98.63, 99.43)	98.67 (97.67, 99.67)	97.08 (96.40, 97.77)

SD=Standard deviation, IQR=Interquartile range, GCT=Maxillary canine gingival margin, ANS=Anal skin, ING=Inguinal canal, EC=Ear canal, BCS=Body condition score, PAL=Palmar pad

From the univariable analysis of the baseline characteristics and rectal temperature, we discovered a significant correlation between the rectal temperature and the age of 0–1 and 1–7 years old (p = 0.001 and 0.006, respectively), but not with other characteristics (Table-2).

**Table-2:** Univariate analysis between baseline characteristics and rectal temperature of cats.

Variables	B (SE)	95% CI	p-value	Variables	B (SE)	95% CI	p-value
Gender				Hair type			
Female	Reference			Short hair	Reference		
Male	−0.03 (0.15)	−0.34, 0.27	0.818	Long hair	0.15 (0.22)	−0.29, 0.59	0.506
				No hair	−0.68 (0.73)	−2.12, 0.76	0.350
Age				Hair color			
0–1 year	Reference			White solid	Reference		
1–7 years	−0.51 (0.16)	−0.82, −0.20	0.001*	Black solid	0.15 (0.31)	−0.46, 0.77	0.626
7–10 years	−0.27 (0.39)	−1.04, 0.49	0.478	Blue solid	0.25 (0.35)	−0.44, 0.94	0.469
>10 years	−1.42 (0.50)	−2.41, −0.42	0.006*	Cream solid	0.38 (0.41)	−0.43, 1.18	0.356
BCS				Red solid	0.08 (0.32)	−0.54, 0.71	0.788
Score 3	Reference			Patterns	0 (0.28)	−0.54, 0.55	0.990
Score <3	−0.22 (0.23)	−0.75, 0.30	0.403	Points	0 (0.33)	−0.66, 0.65	0.992
				No hair	−0.62 (0.77)	−2.14, 0.90	0.421
Score >3	−0.09 (0.16)	−0.42, 0.23	0.563				

* p ≤ 0.05, that age was a confounder, and it was used in the multivariate analysis for the correlation of rectal temperature and infrared temperature with adjusted by baseline characteristics, SE=Standard error, BCS=Body condition score, CI=Confidence interval

Univariable analysis of infrared temperature showed significant correlations between GCT, ANS, ING, and EC and rectal temperature in the cats (p = 0.001, 0.001, 0.066, and 0.012, respectively) (Table-3). Predictor areas with p ≤ 0.2 were used for stepwise forward selection for the final model to predict rectal temperature adjusted by baseline characteristics.

**Table-3:** Univariate analysis of infrared temperature as a predictor area and rectal temperature of cats.

Area	Infrared temperature (°F)	B (SE)	95% CI	p-value

	Mean (SD)			
GCT	98.31 (0.60)	0.40 (0.12)	0.16, 0.64	0.001*
ANS	98.19 (0.71)	0.34 (0.10)	0.14, 0.55	0.001*
ING	97.35 (0.58)	−0.24 (0.13)	−0.49, 0.02	0.066*
EC	98.31 (0.76)	0.25 (0.10)	0.05, 0.44	0.012*
PAL	97.05 (0.42)	0.13 (0.18)	−0.22, 0.49	0.459

* p ≤ 0.2, that predictor areas were used in stepwise forward selection for the final model, and predictor areas with p ≤ 0.05 were used in the next multivariable analysis of rectal temperature and infrared temperature, adjusted by baseline characteristics, SD=Standard deviation, GCT=Maxillary canine gingival margin, ANS=Anal skin, ING=Inguinal canal, EC=Ear canal, PAL=Palmar pad, SE=Standard error, CI=Confidence interval

The multivariable analysis of the predictor area and baseline characteristics of cats is presented in Table-4. The infrared temperatures of cats aged 0–1 year were significantly correlated with those of cats aged 1–7 years in GCT and ANS (p = 0.002 and 0.03) and in ANS with cats aged 7–10 years (p = 0.022). Regarding the correlations between BCSs and infrared temperature, cats with BCS <3 or >3 recorded a lower temperature than cats with BCS of 3 in the ANS (p = 0.02 and 0.045, respectively) and ING regions (BCS >3, p < 0.001). The mean infrared temperature of short-haired cats in the ING tended to be higher than the infrared temperature of long-haired cats by 0.3°F (p = 0.015) but lower than no-hair cats (p < 0.001). The multivariable analysis also showed a significant effect of different hair colors and body regions on the infrared temperature of the cats. In particular, the solid white cat showed lower temperatures than the blue solid cat in GCT (p < 0.05), solid red cat in ANS and EC (p < 0.05), pattern cat in ANS and ING (p < 0.05), and color point cat in ANS (p < 0.05).

**Table-4:** Multivariate analysis of the predictor area and baseline characteristics of cats.

Variables	GCT			ANS			ING			EC		
			
	B (SE)	95% CI	p-value	B (SE)	95% CI	p-value	B (SE)	95% CI	p-value	B (SE)	95% CI	p-value
Gender												
Female	Reference			Reference			Reference			Reference		
Male	0.03 (0.10)	−0.17, 0.23	0.760	−0.11 (0.11)	−0.33, 0.12	0.343	0.01 (0.09)	−0.17, 0.19	0.900	0.06 (0.13)	−0.20, 0.31	0.661
Age												
0–1 year	Reference			Reference			Reference			Reference		
1–7 years	−0.32 (0.11)	−0.52, −0.12	0.002*	−0.25 (0.11)	−0.48, −0.02	0.030*	0.05 (0.09)	−0.13, 0.23	0.603	−0.23 (0.13)	−0.49, 0.02	0.074
7–10 years	0.13 (0.24)	−0.02, 0.93	0.591	−0.63 (0.27)	−1.18, −0.09	0.022*	0.35 (0.22)	−0.07, 0.78	0.104	0.18 (0.31)	−0.42, 0.79	0.554
>10 years	−0.13 (0.31)	−0.42, 0.81	0.675	−0.53 (0.35)	−1.23, 0.16	0.133	0.04 (0.28)	−0.51, 0.59	0.891	−0.53 (0.40)	−1.31, 0.26	0.186
BCS												
Score 3	Reference			Reference			Reference			Reference		
Score <3	−0.09 (0.16)	−0.41, 0.23	0.574	−0.43 (0.18)	−0.80, −0.07	0.020*	−0.12 (0.14)	−0.41, 0.17	0.412	−0.22 (0.21)	−0.63, 0.18	0.277
Score >3	0.20 (0.11)	−0.01, 0.41	0.065	−0.24 (0.12)	−0.48, 0	0.045*	−0.38 (0.09)	−0.57, −0.19	<0.001*	0.05 (0.13)	−0.22, 0.31	0.725
Hair type												
Short hair	Reference			Reference			Reference			Reference		
Long hair	0.05 (0.13)	−0.22, 0.32	0.720	−0.18 (0.15)	−0.48, 0.13	0.244	−0.30 (0.12)	−0.54, −0.06	0.015*	−0.31 (0.18)	−0.65, 0.03	0.075
No hair	0.19 (0.45)	−1.07, 0.69	0.675	0.28 (0.51)	−0.72, 1.28	0.586	1.93 (0.40)	1.14, 2.72	<0.001*	0.45 (0.57)	−0.67, 1.57	0.428
Hair color												
White solid	Reference			Reference			Reference			Reference		
Black solid	0.06 (0.18)	−0.30, 0.42	0.739	0.21 (0.21)	−0.20, 0.62	0.309	0.13 (0.16)	−0.20, 0.45	0.440	0.16 (0.23)	−0.31, 0.61	0.516
Blue solid	0.43 (0.21)	0.03, 0.84	0.037*	0.07 (0.23)	−0.39, 0.54	0.748	−0.06 (0.18)	−0.43, 0.30	0.743	0.09 (0.26)	−0.43, 0.60	0.744
Cream solid	0.35 (0.24)	−0.12, 0.82	0.142	0.22 (0.27)	−0.31, 0.75	0.413	0.23 (0.21)	−0.18, 0.65	0.272	0.22 (0.30)	−0.37, 0.81	0.468
Red solid	0.20 (0.19)	−0.18, 0.58	0.299	0.52 (0.22)	0.10, 0.95	0.017*	0.24 (0.17)	−0.09, 0.58	0.159	0.49 (0.24)	0.01, 0.97	0.043*
Patterns	0.27 (0.17)	−0.07, 0.61	0.124	0.47 (0.20)	0.08, 0.86	0.017*	0.33 (0.15)	0.03, 0.64	0.034*	0.32 (0.22)	−0.11, 0.76	0.142
Points	0.16 (0.20)	−0.23, 0.55	0.431	0.46 (0.22)	0.02, 0.91	0.041*	0.16 (0.18)	−0.19, 0.51	0.380	0.27 (0.25)	−0.22, 0.77	0.283
No hair	N/A	N/A	N/A	N/A	N/A	N/A	N/A	N/A	N/A	N/A	N/A	N/A

* p ≤ 0.05, that baseline characteristics were confounders and were used in multivariable analysis for correlation of rectal temperature and infrared temperature adjusted by baseline characteristics. N/A is not applicable data, GCT=Maxillary canine gingival margin, ANS=Anal skin, ING=Inguinal canal, EC=Ear canal, BCS=Body condition score, SE=Standard error, CI=Confidence interval

The multivariable analysis for the pre-prediction model of rectal temperature based on the infrared temperature from a predictor region adjusted for baseline characteristics is presented in Table-5. The infrared temperatures of the ANS, GCT, and EC were used to predict rectal temperature (p < 0.01 for ANS and GCT, and p < 0.05 for EC).

**Table-5:** Multivariate analysis for regression model of rectal temperature prediction using infrared temperature as a predictor area adjusted by baseline characteristics of cats.

Variables	ING			ANS			GCT			EC		
			
	B (SE)	95% CI	p-value	B (SE)	95% CI	p-value	B (SE)	95% CI	p-value	B (SE)	95% CI	p-value
Constant	123.57 (14.11)	95.71, 151.43		70.60 (11.00)	48.88, 92.32		68.34 (12.38)	43.89, 92.78		81.53 (9.82)	62.14, 100.92	
Predictor area	−0.23 (0.14)	−0.52, 0.05	0.112	0.31 (0.11)	0.09, 0.53	0.006*	0.33 (0.13)	0.08, 0.58	0.009*	0.20 (0.10)	0, 0.40	0.048*
Age												
0–1 year	Reference			Reference			Reference			Reference		
1–7 years	−0.58 (0.17)	−0.91, −0.24	0.001	−0.49 (0.17)	−0.83, −0.16	0.004	−0.46 (0.16)	−0.78, −0.13	0.006	−0.50 (0.16)	−0.82, −0.18	0.003
7–10 years	−0.32 (0.41)	−1.12, 0.48	0.434	−0.13 (0.40)	−0.93, 0.66	0.743	−0.35 (0.39)	−1.12, 0.42	0.370	−0.30 (0.39)	−1.07, 0.47	0.442
>10 years	−1.63 (0.52)	−2.67, −0.60	0.002	−1.41 (0.52)	−2.43, −0.38	0.007	−1.51 (0.51)	−2.51, −0.51	0.003	−1.41 (0.51)	−2.43, −0.39	0.007
BCS												
Score 3	Reference			Reference			NCE	NCE	NCE	NCE	NCE	NCE
Score <3	−0.18 (0.27)	−0.72, 0.35	0.500	0.04 (0.27)	−0.50, 0.57	0.887						
Score >3	0.03 (0.18)	−0.32, 0.39	0.854	0.20 (0.17)	−0.14, 0.54	0.257						
Hair type												
Short hair	Reference			NCE	NCE	NCE	NCE	NCE	NCE	NCE	NCE	NCE
Long hair	0.23 (0.23)	−0.22, 0.69	0.312									
No hair	−0.33 (0.80)	−1.90, 1.24	0.682									
Hair color												
White solid	Reference			Reference			Reference			Reference		
Black solid	0.26 (0.30)	−0.34, 0.86	0.400	0.17 (0.30)	−0.42, 0.77	0.568	0.23 (0.30)	−0.36, 0.82	0.447	0.23 (0.30)	−0.36, 0.83	0.445
Blue solid	0.39 (0.34)	−0.28, 1.06	0.256	0.33 (0.33)	−0.33, 0.99	0.331	0.23 (0.34)	−0.43, 0.90	0.490	0.36 (0.34)	−0.31, 1.02	0.292
Cream solid	0.42 (0.40)	−0.36, 1.20	0.289	0.24 (0.39)	−0.53, 1.01	0.543	0.23 (0.39)	−0.54, 1.00	0.562	0.30 (0.39)	−0.48, 1.07	0.450
Red solid	0.43 (0.40)	−0.20, 1.06	0.180	0.15 (0.32)	−0.48, 0.78	0.646	0.27 (0.31)	−0.34, 0.89	0.380	0.24 (0.32)	−0.38, 0.87	0.448
Patterns	0.15 (0.27)	−0.39, 0.70	0.572	−0.10 (0.27)	−0.64, 0.44	0.722	−0.01 (0.26)	−0.53, 0.51	0.969	0.02 (0.27)	−0.50, 0.55	0.925
Points	0.15 (0.32)	−0.49, 0.79	0.643	−0.05 (0.32)	−0.69, 0.59	0.873	0.07 (0.32)	−0.55, 0.70	0.813	0.08 (0.32)	−0.55, 0.71	0.806
No hair	N/A	N/A	N/A	−0.92 (0.72)	−2.37, 0.54	0.215	−0.72 (0.73)	−2.16, 0.72	0.326	−0.86 (0.74)	−2.32, 0.60	0.246

* p ≤ 0.05, that infrared temperature form predictor areas could predict rectal temperature with adjusted by baseline in the regression model. N/A=Not applicable data. NCE=No-confounder effect, GCT=Maxillary canine gingival margin, ANS=Anal skin, ING=Inguinal canal, EC=Ear canal, BCS=Body condition score, SE=Standard error, CI=Confidence interval

The pre-prediction models using ANS (Adjusted R^2^ = 0.0866, p = 0.0074) are: Rectal temperature (°F) = 70.60285 + (0.3105509 × infrared temperature [°F] on ANS) + A + B + C.

Where A is 0 if age <1 year, −0.4949252 if age 1–7 years, −0.1323931 if age 7–10 years, or −1.40914 if age >10 years; B is 0 if BCS (full score is 5) is 3, 0.0387731 if BCS <3, or 0.196651 if BCS >3; and C is 0 if hair color is white solid, 0.1729044 if black solid, 0.326424 if blue solid, 0.2379375 if cream solid, 0.1466407 if red solid, 0.0977855 if pattern, 0.0517731 if point, or 0.9179764 if no hair.

The pre-prediction models using GCT (Adjusted R^2^ = 0.0893, p = 0.0042) are: Rectal temperature (°F) = 68.33647 + (0.3332861 × infrared temperature [°F] on GCT) + A + C.

Where A is 0 if age <1 year, −0.4586486 if age 1–7 years, −0.3496701 if age 7–10 years, or −1.512057 if age >10 years; and C is 0 if hair color is white solid, 0.2282577 if black solid, 0.2343358 if blue solid, 0.2265719 if cream solid, 0.2736995 if red solid, 0.0103138 if pattern, 0.0751761 if point, or 0.7207356 if no hair.

The pre-prediction models based on EC (Adjusted R^2^ = 0.0734, p = 0.0118) are: Rectal temperature (°F) = 81.53249 + (0.198937 × infrared temperature [°F] on EC) + A + C.

Where A is 0 if age <1 year, −0.5014771 if age 1–7 years, −0.3015112 if age 7–10 years, or −1.41138 if age >10 years; and C is 0 if hair color is white solid, 0.2312166 if black solid, 0.3560666 if blue solid, 0.2963913 if cream solid, 0.2412105 if red solid, 0.0252792 if pattern, 0.0788468 if point, or 0.8609329 if no hair.

**Figure-1:**
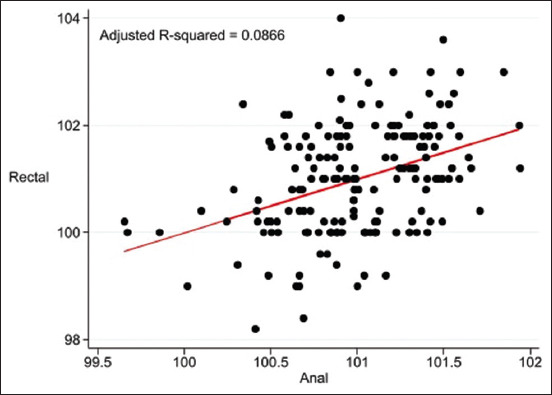
Scatter plot of temperature (°F) from pre-prediction model of infrared temperature on anal skin and rectal temperature adjusted for baseline characteristics of cats.

**Figure-2:**
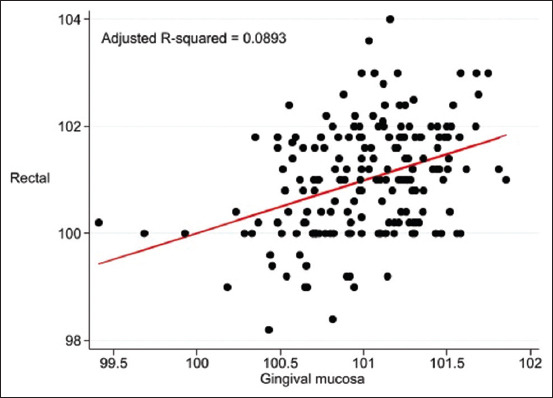
Scatter plot of temperature (°F) from pre-prediction model infrared temperature on maxillary gingival mucosa above canine teeth and rectal temperature adjusted by baseline characteristics of cats.

**Figure-3:**
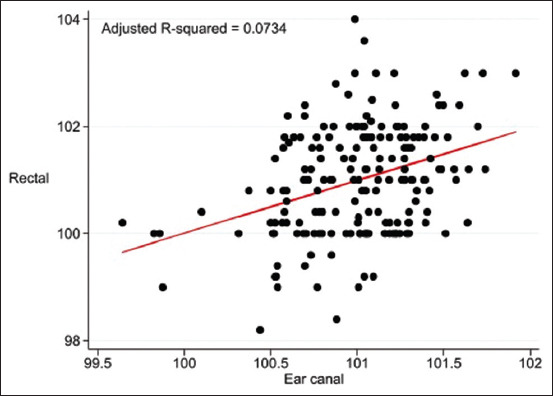
Scatter plot of temperature (°F) from the pre-prediction model of infrared temperature in the ear canal and rectal temperature adjusted for baseline characteristics of cats.

The calibration data from the ANS, GCT, and EC for the pre-prediction model are presented as scatter plots in Figures-1, 2, and 3, respectively.

After forward stepwise selection, no correlation coefficients were observed between the infrared temperature on EC, GCT, and rectal temperature (p > 0.05). As a result, the final prediction model of rectal temperature using an infrared thermometer combined ANS and ING (Table-6). The model (Adjusted R^2^ = 0.1276, p = 0.0008) is:

**Table-6:** Multivariate analysis of the correlation between predictor areas adjusted for baseline characteristics and rectal temperature after stepwise regression analysis.

Characteristic	B (SE)	95% CI	p-value	Characteristic	B (SE)	95% CI	p-value
Constant	97.93 (15.33)	67.67, 128.20					
Area				Hair type			
ANS	0.42 (0.11)	0.19, 0.65	<0.001	Short hair	Reference		
ING	−0.39 (0.15)	−0.68,−0.10	0.008	Long hair	0.27 (0.22)	−0.17, 0.71	0.234
				No hair	−0.15 (0.77)	−1.67, 1.37	0.844
Age				Hair color			
0–1 year	Reference			White solid	Reference		
1–7 years	−0.47 (0.17)	−0.80, −0.14	0.006	Black solid	0.17 (0.30)	−0.41, 0.76	0.554
7–10 years	−0.01 (0.40)	−0.80, 0.79	0.983	Blue solid	0.32 (0.33)	−0.32, 0.97	0.323
>10 years	−1.41 (0.51)	−2.42,−0.40	0.006	Cream solid	0.35 (0.38)	−0.40, 1.11	0.360
BCS				Red solid	0.24 (0.31)	−0.38, 0.85	0.452
Score 3	Reference			Patterns	−0.02 (0.27)	−0.55, 0.51	0.937
Score <3	−0.02 (0.27)	−0.54, 0.51	0.949	Points	−0.04 (0.32)	−0.67, 0.58	0.894
Score >3	0.09 (0.17)	−0.26, 0.43	0.615	No hair	N/A	N/A	N/A

N/A=Not applicable data, ANS=Anal skin, ING=Inguinal canal, SE=Standard error, CI=Confidence interval, BCS=Body condition score

Rectal temperature (°F) = 97.93493 + (0.4207463 × infrared temperature [°F] in ANS) + (−0.3925218 × infrared temperature [°F] in ING) + A + B + C + D.

Where A is 0 if age of cat <1 year, −0.4674662 if age 1–7 years, −0.0083397 if age 7–10 years, or −1.410084 if age >10 years; B is 0 if BCS of cat 3 year, −0.0172102 if BCS <3 year, or 0.0883376 if BCS >3 year; C is 0 if hair color is white solid, 0.1756309 if black solid, 0.3255063 if blue solid, 0.3513499 if cream solid, 0.2362285 if red solid, 0.0211786 if pattern, or 0.0422609 if point; and D is 0 if hair type is shot-hair, −0.1516942 if no hair, and 0.2663488 if long hair.

**Figure-4:**
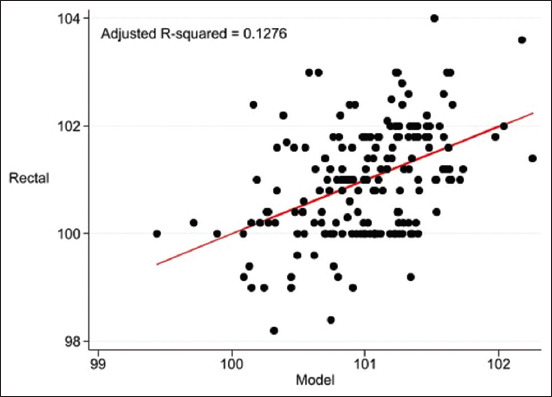
Scatter plot of temperature (°F) from final prediction model using infrared temperature of anal skin combined with inguinal area and adjusted by baseline characteristics of cats.

The calibration data from the ANS and ING for the final prediction model are presented as scatter plot in Figure-4.

## Discussion

This study was designed to predict rectal temperature using an infrared thermometer measured across various body regions, adjusted according to baseline characteristics. According to a study by Mota-Rojas *et al*. [29] on physiological and behavioral changes in mammals, stressful stimuli can influence infrared temperature. To address this limitation, aggressive cats were excluded from the study. Moreover, this study did not gather information regarding cat owners because no significant association was found between cat bodyweight, rural-urban settings, and socioeconomic status [30]. A comparison of the infrared temperature measurements of the different body areas and the rectal temperature showed that the infrared thermometer recorded lower temperature values than the rectal temperature. The results of this study are in accordance with a previous study by Barton *et al*. [31] using an infrared thermometer in different animals [31]. In addition, our study and McNicholl *et al*. [32] and Boehm and Miller [33] reported almost no correlation between sex, rectal temperature, and infrared temperature. Further research evaluating the effect of sex on rectal and infrared temperature performance will provide valuable insights.

Age difference affected the rectal and infrared temperatures of the ANS and GCT in this study (Tables-2 and 4). This finding contradicts a previously reported finding by Kotrba *et al*. [34] in even-toed ungulate species that the infrared temperatures of younger animals were not significantly different from those of older animals.

There were significant correlations between rectal temperature and infrared temperature in the ANS, EC, and GCT, but not in PAL (Tables-3 and 5). It can be explained that small blood vessels near the surface of the skin yield higher temperature readings than other areas like dorsal metacarpal veins [35]. Many studies have been conducted to validate the correlation between non-contact handheld infrared thermometers for recording body and rectal temperatures; Rectal temperature was not significantly correlated with the temperature of the pinna in dogs and cats [31] and the EC in dogs and cats [1, 16, 36], but was more precise in dogs’ auricle and gum [21, 37, 38], and the eardrum of piglets [39]. This difference could be due to physiological temperature differences in the ear region among species. After adjusting for baseline variables, the infrared temperature of the inguinal area was not significantly correlated with the rectal temperature (Table-5). The obtained result is consistent with a previous study by Giannetto *et al*. [40] in cats in which the infrared temperature measurements of the jugular, shoulder, rib, flank, and inner thigh regions showed poor agreement with the rectal temperature.

Interestingly, the opposite tendency was found in piglets, in which the infrared temperature measurements at the inner thigh and abdomen were highly correlated with the core temperature [39]. In addition, a recent study by Cugmas *et al*. [38] found that the infrared temperature in the inguinal area of dogs was significantly correlated with rectal temperature. However, our study found that the infrared temperatures of the ANS, EC, and GCT could be used to predict rectal temperature in cats adjusted for baseline characteristics. This result corresponded to a previous study by Lukkanawaraporn *et al*. [41] in dogs in which the infrared temperatures on the surface and auricle were the most appropriate predictors of rectal temperature.

The BCS of cats in this study was significantly correlated with the infrared temperature of the ANS and inguinal area after adjusting for other characteristics (Table-4). Our findings indicate that normal cats (BCS = 3) had a higher infrared temperature than underweight cats (BCS <3) in the ANS and obese cats (BCS >3) in both the ANS and ING. This finding partially agrees with previously reported findings in dogs showing a lower body temperature in small breeds than in large breeds [21]. In contrast, a previous study by Christopherson and Young [42] reported higher skin temperatures in larger animals, which might be attributable to the amount of subcutaneous fat, hair coat thickness, and ambient environment. Therefore, this should be further investigated.

The infrared temperature recorded in the inguinal area was significantly correlated with hair type (Table-4). Our study observed the highest infrared temperature in non-haired cats, followed by short-haired and long-haired cats. This phenomenon could be due to the regulation of skin temperature based on the interaction between the skin microcirculation and the external environment [23]. Close agreement has been reported for dogs, in which long-haired and double-coat dogs have lower mean surface temperatures than short-haired and curly-coat dogs. However, no significant effect of coat types on evaluating differences in body surface temperature was demonstrated in the study [20]. Hair color also had a significant correlation with infrared temperature, with cats of solid white color developing lower infrared temperatures than those of solid blue (in GCT), solid red (in ANS and EC), pattern (in ANS), and point (in ANS) colored cats (Table-4). This finding agrees with findings in other species, in which it was revealed that white animals can regulate body temperatures at lower temperatures than dark-colored animals [32].

In the single-predictor measurements, ANS, EC, and GCT were used as indicators for predicting rectal temperature (Table-5). After forward stepwise selection, the infrared temperatures of the ING and ANS were significantly correlated with rectal temperature and were adjusted according to baseline characteristics (Table-6). The adjusted R-squared values of the pre-prediction model of infrared temperature on anal skin and rectal temperature (Figure-1), maxillary gingival mucosa above canine teeth and rectal temperature (Figure-2), EC and rectal temperature (Figure-3), and final prediction model by infrared temperature on ANS combined with inguinal area (Figure-4) were low (0.0866 or 8.7%, 0.0893 or 8.9%, 0.0734 or 7.3%, and 0.1276 or 12.8%, respectively), that were be this study limitation.

The limitation of this study was the lack of cats 7 years of age and above. Small sample sizes may cause selection bias and affect the lack of significant results in these categories. Moreover, this study was not designed to collect environmental data. As stated previously by Rekant *et al*. [25] and Vainionpää *et al*. [43], the ambient environment, such as room temperature and relative humidity, might affect body skin temperature and can lead to misinterpretation, previous studies by Kwon and Brundage [20] on dogs found that environmental conditions did not significantly affect rectal and body temperatures. However, this study is the first to design a prediction model for rectal temperature prediction using an infrared thermometer. In future studies, we have to develop a prediction model from this model by another factor or use it in external validation to test the validity and reliability of this model for assessing its practical usefulness.

## Conclusion

This study demonstrated that the infrared temperatures in the ANS combined with the ING area using a non-contact infrared thermometer may provide good parameters for predicting rectal temperature in cats under the effects of age, BCS, hair type, and hair color. The infrared temperature measured from the ANS, EC, or GCT showed correlations for rectal temperature prediction based on one region. However, the adjusted R-squared values of all models were low; thus, the predictive model will need to be developed and used to test validity and reliability with an external study group to assess their practical usefulness.

## Authors’ Contributions

NN and TJ: Designed and conducted the study. NN, SC, and TJ: Interpreted the results. NN: Drafted the manuscript. SC: Revised and finalized the manuscript for submission. MS, RM, and NN: Supervised the clinical techniques. All authors have read, reviewed, and approved the final manuscript.
